# Giant Faraday Rotation through Ultrasmall Fe^0^ *_n_* Clusters in Superparamagnetic FeO‐SiO_2_ Vitreous Films

**DOI:** 10.1002/advs.201600299

**Published:** 2016-12-05

**Authors:** Yuko Nakatsuka, Kilian Pollok, Torsten Wieduwilt, Falko Langenhorst, Markus A. Schmidt, Koji Fujita, Shunsuke Murai, Katsuhisa Tanaka, Lothar Wondraczek

**Affiliations:** ^1^ Department of Material ChemistryGraduate School of EngineeringKyoto UniversityKatsuraNishikyo‐kuKyoto615‐8510Japan; ^2^ Otto Schott Institute of Materials ResearchUniversity of JenaFraunhoferstr. 607743JenaGermany; ^3^ Institute of GeosciencesUniversity of JenaCarl‐Zeiss‐Promenade 1007745JenaGermany; ^4^ Leibnitz Institute of Photonic TechnologyAlbert‐Einstein‐Str. 907745JenaGermany

**Keywords:** amorphous oxides, magnetooptics, ultrasmall metallic particles

## Abstract

Magnetooptical (MO) glasses and, in particular, Faraday rotators are becoming key components in lasers and optical information processing, light switching, coding, filtering, and sensing. The common design of such Faraday rotator materials follows a simple path: high Faraday rotation is achieved by maximizing the concentration of paramagnetic ion species in a given matrix material. However, this approach has reached its limits in terms of MO performance; hence, glass‐based materials can presently not be used efficiently in thin film MO applications. Here, a novel strategy which overcomes this limitation is demonstrated. Using vitreous films of *x*FeO·(100 − *x*)SiO_2_, unusually large Faraday rotation has been obtained, beating the performance of any other glassy material by up to two orders of magnitude. It is shown that this is due to the incorporation of small, ferromagnetic clusters of atomic iron which are generated *in line* during laser deposition and rapid condensation of the thin film, generating superparamagnetism. The size of these clusters underbids the present record of metallic Fe incorporation and experimental verification in glass matrices.

## 1 Introduction

**Figure 1:**
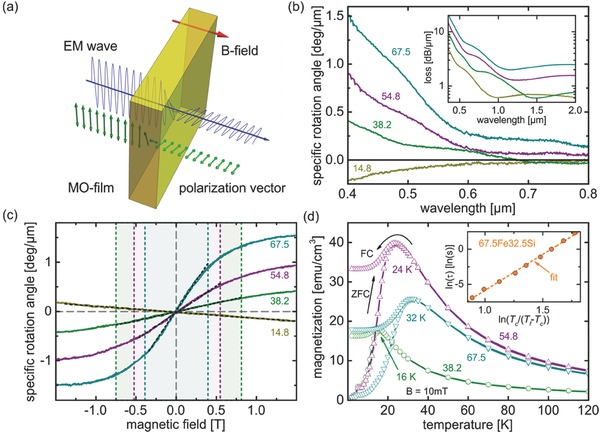
Prominent Faraday rotation in vitreous iron silicate layers. a) Principle of the Faraday effect: Rotation of the polarization state of a linearly polarized electromagnetic wave passing through a film experiencing a longitudinal static magnetic field (blue: electromagnetic wave, green arrows: polarization vector, yellow: film, red arrow: direction of magnetic field). In (b) the specific rotation angle in vitreous layers FeO‐SiO_2_ is shown as a function of wavelength at a constant field of 1.5 T for different molar fractions of FeO (labels), revealing very high rotation for FeO exceeding 38.2 mol%. The corresponding spectrum of optical attenuation is shown in the inset. (c) depicts the field dependence of the rotation angle at a wavelength of 400 nm, highlighting the field regimes of linear dependence for which an effective Verdet constant was calculated (black dashed line, see text for details). The magnetization data shown in (d) are replotted for reference from Ref. 25. Here, magnetization is shown for a DC field of 0.01 T, for field cooled (FC) and zero field cooled (ZFC) specimen. The inset shows the frequency dependence of the temperature of freezing, *T*_f_, reduced over the extrapolated freezing temperature for infinite relaxation time, *T*_c_.

Magnetooptical (MO) effects play a key role in optical information processing, data coding and, more generally, laser technology.^1^ Here, the Faraday effect is one of the most widely employed magnetooptical effects (schematically shown in **Figure** **1**a): when linearly polarized light passes through a transparent longitudinally magnetized material, the plane of polarization rotates as a function of the amplitude of the externally applied magnetic field. Since the Faraday effect is an optically nonreciprocal phenomenon, it is used to construct optical isolators, sensors, diodes, and switches. In many of these applications, Y_3_Fe_5_O_12_ (YIG),^2^ Bi_3_Fe_5_O_12_ (BIG),^3^ and Tb_3_Ga_5_O_12_ 4 crystals are the present benchmark materials because of their large Faraday effect, especially in the infrared spectral region. Together with high optical transparency, this provides efficient Faraday rotation at low optical loss.

However, due to high optical loss,^2^, ^5^ neither YIG nor BIG single‐crystals are suitable for applications in wavelength regimes other than the infrared, in particular, in the visible (Vis) or even UV spectral ranges. In addition, their use is restricted to a relatively small number of substrate materials,^6^, ^7^ and also possibilities for implementation with fiber optical devices are rather limited.^8^ These issues present very strict limitations which can only be overcome with alternative material solutions. For one, besides telecommunication, spectral ranges beyond the infrared play a very important role, e.g., in magnetooptical coding or for oscillation stabilization in blue lasers,^9^ Furthermore, combination of a MO device with oxide substrates such as vitreous silica or others, e.g., through fiber splicing or direct deposition, is key to almost any specific application in the above areas. Crystallinity of the MO material is a major drawback in this context. Other materials have therefore been proposed and applied, such as rare‐earth (Eu^2+^, Tb^3+^) containing glasses, where the paramagnetic rare‐earth ion is supposed to ensure high magnetooptical activity.^10^, ^11^, ^12^, ^13^ The development of such glasses has been following a simple strategy, aiming for incorporation of an as high as possible fraction of the paramagnetic ion species without triggering destructive effects such as clustering, phase separation, or material crystallization.^14^, ^15^ The joint basis of these approaches is that glasses provide extremely high optical homogeneity, very high surface quality and, in most cases,^16^ structural isotropy which simplifies implementation and operation of the respective optical devices. In addition, compared to crystalline candidates, glassy materials provide the technological advantage of universal process ability which enables forming into a very broad variety of shapes, including the ability for coating onto virtually any kind of substrate material. Another significant advantage over their crystalline counterparts is that their physical properties can usually be tuned continuously through changing the composition. However, the absence of structural order leads to an inherent downside, i.e., a usually significantly lower rotation angle per unit sample thickness as compared to crystalline materials. This has led to the present limit in the achievable Verdet constant of glasses of roughly −120 rad T^−1^ m^−1^ at wavelengths around 630 nm.^14^ In most cases, such low rotation efficiency requires that glasses are used in bulk form in order to obtain a total rotation angle which is comparable to that of the crystalline benchmark materials. This, on the other side, stands in sharp contrast to the recently increasing demand for more compact integrated device architectures. Thus, new routes toward notably enhanced Faraday rotation in vitreous matrices are highly desired, and some approaches have been proposed in this context. For example, the use of magnetophotonic crystals,^17^ combination with surface plasmon resonance,^18^, ^19^, ^20^, ^21^ or inclusion of magnetic nanoparticles into glass matrices^22^, ^23^ have been considered recently. However, none of these approaches targets the chemistry of the MO material as such. Rather, they are all usually associated with significantly more complicated processing and also significantly higher optical loss, making widespread application rather unlikely.

The present approach provides an alternative which is overcoming the noted limitations, enabling record Faraday rotation in a vitreous material and, hence, thin‐film application. This is achieved by generating ferromagnetic clusters of elemental iron which are, during a rapid reactive gas‐phase condensation process, incorporated into a glassy matrix of iron silicate to form a homogeneous, superparamagnetic layer of FeO‐SiO_2_:$Fe_{\;\;n}^{0}$. This route can be generalized as a design strategy for super‐efficient vitreous Faraday rotators.

## 2 Results and Discussion

### 2.1 Magnetooptical Properties and Faraday Rotation of FeO‐SiO_2_ Films

The magnitude of magnetooptical activity of a material is often expressed through the Verdet constant *V*, which provides a straightforward measure for the Faraday rotation angle θ as a function of the applied magnetic field with strength *B*, and the geometrical path length *d* of linearly polarized light passing through the rotator material, θ = *VBd*. This simplistic formalism, however, does not apply to ferromagnetic materials in which magnetic saturation is approached with increasing magnetic field strength, and θ becomes independent on field strength. The Verdet constant is therefore often used only as a comparative estimate for MO performance. For the present films of *x*FeO·(100 − *x*)SiO_2_, the specific rotation angles are shown directly as a function of wavelength for a magnetic field of 15 kOe in Figure 1b. The spectral optical attenuation is shown for reference in the inset of Figure 1b. For a nominal iron oxide fraction of *x* = 67.5, 54.8, 38.2, and 14.8, at a wavelength of 633 nm, we obtain rotation values of 4110, 1400, 910, and −680 rad m^−1^, respectively. Especially for the higher contents of FeO, these absolute values exceed the previous benchmarks of Eu^2+^ or Tb^3+^‐containing glasses by up to two orders of magnitude. For example, the Verdet constant of 58.0EuO·12.0Al_2_O_3_·20.0B_2_O_3_·10.0SiO_2_ is about −300 rad T^−1^ m^−1^ at 633 nm,^11^ and that of 17GeO_2_·23B_2_O_3_·32Al_2_O_3_·28Tb_2_O_3_ is −120 rad T^−1^ m^−1^.^14^ Instead, similarly high values have presently been known only for ferrimagnetic crystals such as YIG. When the composition of the films is shifted to the silica‐rich side, the value of *V* rapidly decreases toward that of similar iron oxide containing paramagnetic glasses. The surprisingly high Faraday rotation is therefore clearly related to the presence of highly paramagnetic ferrous iron, but its full magnitude cannot be explained on the basis of Fe^2+^ alone. That is, ferrous phosphate glasses have previously been reported with Verdet constants of around −60 rad T^−1^ m^−1^ at a wavelength of 405 nm,^24^ i.e., two orders of magnitude below the values of the present observation. The field dependence of Faraday rotation is shown in Figure 1c. Up to a field strength of about 0.4 T (≈4 kOe), a roughly linear scaling is observed for all investigated materials. This allows for calculation of an effective Verdet constant within this field regime which can be used for comparative purposes (**Table** **1**). At field strength higher than *B*_sat_ indicated in Table 1, Faraday saturation sets‐in, in accordance with magnetization data of similar materials.^25^

**Table 1:** Real part *n* and imaginary part *k* of the refractive index, effective Verdet constant at 400 nm, *V*_ef_, and saturation onset *B*_sat_ of the studied films. The imaginary parts of the refractive indices have been determined by two independent methods, i.e., through ellipsometry (*k*_el_) and from the optical absorption spectra (*k*_abs_ = α_abs_·λ/(2π))

Sample	*n*	*k*_el_	*k*_abs_	*V*_ef_ [deg (µmT)^−1^]	*B*_sat_ [T]
*x* = 14.8	1.647	0.042	0.037	−0.122	1.5
*x* = 38.2	1.743	0.064	0.075	0.360	0.8
*x* = 54.8	1.88	0.097	0.096	1.090	0.5
*x* = 67.5	2.00	0.131	0.122	2.417	0.4

As the magnitude of the Faraday effect in the present materials can clearly not be traced to Fe^2+^ species, magnetization data are reinspected more closely (Figure 1d). Comparison of magnetization as a function of temperature for field cooled and zero field cooled samples reveals a spin‐melting process in the temperature regime of ≈16–32 K, depending on sample composition and observation time, and significantly reduced magnetization at higher temperature. This is indicative for superspin glass freezing, caused by diverging timescales of observation and Néel relaxation of spins in small clusters with intercluster interaction. The frequency dependence of this relaxation process, shown in the inset of Figure 1d as the function of relaxation time versus the reduced freezing temperature follows a scaling law with the critical exponent of *zν* = 10.3 for *x* = 67.5, confirming the superspin glass transition. In the previous work, the temperature dependence of the inverse magnetizations was analyzed by Curie–Weiss law, and the effective number of Bohr magneton, *M*_B_, of 28, 34, and 29 was obtained for *x* = 38.2, 54.8, and 67.5,^25^ respectively. These values are much larger than the theoretical spin‐only value of Fe^2+^, 4.9, which lets anticipate that each cluster has ferromagnetic ordering. As an intermediate conclusion at this point, it is therefore assumed that the extreme degree of Faraday rotation is related to the presence of small ferromagnetic cluster species.

### 2.2 X‐Ray Photoelectron Spectroscopy (XPS) and X‐Ray Absorption Near Edge Structure (XANES) Analyses of Iron Valence State

**Figure 2:**
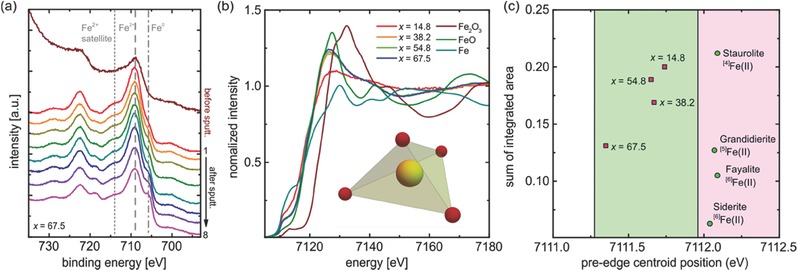
Spectroscopic analyses of iron precipitation in vitreous FeO‐SiO_2_ layers. a) Exemplary XPS data for a sample with FeO content of 67.5 mol% before sputtering and with increasing sputtering depth, where one sputtering cycle (labels) corresponds to ≈1 nm of sputtering depth. (b) presents XANES spectra, together with reference scans on metallic Fe, FeO, and Fe_2_O_3_, evidencing the presence of Fe^2+^ and Fe^0^, and an average coordination change from ^IV^Fe^2+^ to ^V^Fe^2+^. (c) summarizes the sums of integrated band areas in comparison to mineral data from Ref. 28.

XPS and XANES analyses were employed as complementary methods to elucidate the atomic state in which the iron species is incorporated into the present material and, thus, to provide further evidence for the chemical origin of the magnitude of Faraday rotation. This assumes that iron is the key element for producing the observed increase in MO performance. An exemplary XPS spectrum is shown for the highest iron content of *x* = 67.5 in **Figure** **2**a, comparing surface and bulk state of the material by examining the thin film in its pristine state and during sputtering. For the nonsputtered state, the binding energies of Fe 2p_3/2_ and Fe 2p_1/2_ and the satellite of Fe 2p_3/2_ are 708.9 and 722.5, and 713.3 eV, respectively. For reference, the Fe 2p_3/2_ band position of analytical grade Fe, FeO, and Fe_2_O_3_, respectively, was determined at 706.8, 709.8, and 711.2 eV.^26^ In the present case, we find that the binding energy of Fe 2p_3/2_ of the sample with *x* = 67.5 exhibits a best match with FeO, indicating that iron is present in its divalent state, Fe^2+^. Upon sputtering, there is a slight shoulder band located at ≈705.8 eV which increases in intensity with increasing sputtering depth (i.e., increasing number of sputtering cycles, where for pure SiO_2_, a sputtering rate of 0.95 nm per sputtering cycle was determined). This latter band is assigned to 2p_3/2_ in metallic iron, Fe^0^. The further band located at ≈718.6 eV which also increases in intensity during sputtering is attributed to 2p_1/2_ in Fe^0^ (with a reference position of 719.8 eV in metallic iron^26^). These observations provide evidence for the presence of metallic iron in the film. At this point, it can however not be fully excluded that this is a result of Fe^2+^ reduction induced by the ion sputtering process. Therefore, independent verification is obtained from XANES analyses (Figure 2b). For the references of Fe_2_O_3_, FeO, and Fe, respectively, we find an absorption edge of 7125.8, 7121.4, and 7110.6 eV. The absorption edges of the thin films are found at 7118.1, 7119.2, 7119.2, and 7119.2 eV for *x* = 14.8, 38.2, 54.8, and 67.5, i.e., best matching that of FeO and partially overlain by contributions from Fe^0^. They are also close to the absorption edge of crystalline iron orthosilicate with *x* = 67.5, fayalite, at 7120.5 eV.^27^ The pre‐edge centroid position is 7111.74, 7111.67, 7111.65, and 7111.35 eV for *x* = 14.8, 38.2, 54.8, and 67.5, respectively. These values are in the lower energy side compared to those of Fe^2+^‐bearing minerals such as staurolite (7112.09 eV), grandidierite (7112.07 eV), siderite (7112.04 eV), fayalite (7112.09 eV), and Fe^2+^‐containing alkali silicate glasses^28^ (Figure 2c). The sum of integrated band areas for *x* = 14.8 is close to that of staurolite, where divalent iron ions occupy tetrahedral lattice sites. This value decreases with increasing iron fraction *x*, providing evidence for an increase in iron coordination number (with fivefold coordination ^V^Fe^2+^, in the grandidierite mineral reference). In a first consideration, this observation is in very good agreement with XPS data in that iron is predominantly present in its divalent form, Fe^2+^. Additional evidence, however, is provided for the further presence of Fe^0^ or metallic iron at increasing extent with increasing iron content. However, as with XPS, this evidence remains ambiguous, here due to eventual photoreduction by high‐energy X‐ray irradiation.^29^

### 2.3 Direct Observation of Iron Clusters

**Figure 3:**
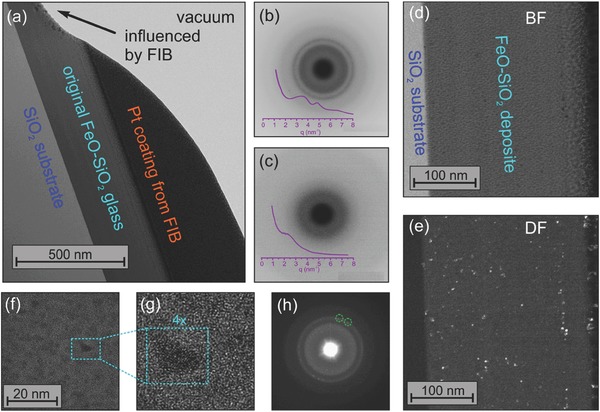
Analytical transmission electron microscopy on vitreous FeO‐SiO_2_. Taking the example of *x* = 67.5, an overview of sample preparation is shown in (a), indicating the locations of further observations on a FIB‐cut specimen. (b) and (c) provide SAED patterns of the deposit layer of FeO‐SiO_2_ and of the substrate, respectively. Bright‐ and composite dark‐field TEM images are given in (d) and (e) (see text for details), and a HR‐TEM closeup is shown in (f)–(h), clearly visualizing the presence of particles with a regular lattice spacing of ≈0.2 nm, and the corresponding SAED pattern with several sharp reflections (exemplarily indicated with circles) originating from metallic iron.

For final evidence of the presence, size and number density of metallic iron particles or clusters, analytical transmission electron microscopy (TEM) was performed. Exemplary TEM images (obtained on sample *x =* 67.5) are shown in **Figure** **3**. A general overview of an ion‐thinned specimen is provided in Figure 3a, showing a cross‐section of the as‐deposited iron silicate thin film on the silica substrate, together with layers of carbon and platinum that were deposited during focused‐ion beam (FIB) sample preparation. The darker appearance of the as‐deposited film is a result of the higher electron absorption cross section of the iron silicate as compared to the silica substrate. Selected area electron diffraction (SAED) on silica substrate and on the iron silicate thin film shows distinct differences, where the film exhibits a more complex diffraction pattern as compared to the glass substrate (which yields only the broad halo of a typical amorphous material (Figure 3b,c). In particular, the SAED pattern taken on the film exhibits an additional, broad ring which reflects ordering at an atomic distance of ≈2 Å, consistent with the lattice spacing of the (110) plane of metallic iron. However, as this spacing is also close to the Fe–O distance in typical iron‐bearing silicate glasses and does therefore not allow for final assignment to metallic iron. More detailed inspection by bright‐ and composite dark‐field (DF) imaging (Figure 3d,e) clearly points to an inhomogeneous film structure as compared to the substrate. In the bright‐field (BF) image, this is not yet unambiguous evidence for the presence of cluster or particle species because similar fluctuations could also be generated by, e.g., variations in sample thickness. However, the very same fluctuations are also visible in the dark field image taken with electrons from the diffraction ring at 2 Å. This excludes thickness fluctuations. Instead, iron nanoparticles which are in diffraction condition in one of the underlying DF images are displayed as bright speckles in Figure 3e. The size (diameter) of these clusters is in the order of 2–3 nm. A closeup of the microstructure is subsequently obtained byhigh‐resolution (HR) TEM (Figure 3f,g). Imaging of the tiny Fe particles is complicated by the fact that they are visualized in projection together with the over‐ and underlying glass. Therefore, the images represent a superposition of information from particles and glass. Despite this, several of the 1–3 nm Fe particles could be directly imaged. Here, an individual particle with a size of about 2 nm was selected for SAED and high‐resolution imaging, indicating a regular lattice spacing of 2.04 Å and a textured diffraction pattern with distinct diffraction spots along the Fe(110) diffraction ring (Figure 3h). Together with the independent confirmations by XPS and XANES, these observations provide clear evidence for the presence of metallic iron in the pulsed laser deposition (PLD)‐deposited layers of FeO‐SiO_2_.

Effective refractive indices *ñ*_ef_ = *n* + *ik* as obtained from ellipsometric analyses of the films are shown in Table 1, where *ñ* is the complex refractive index, *n* is its real part, and *k* is the extinction coefficient. The observed values are larger than expected for a typical oxide glass, i.e., *n* = 1.509 and *k* = 8 × 10^−8^ for SiO_2_ glass.^30^ We however believe that the presented index values are correct since the ellipsometric measurements yields an imaginary of the refractive index *k*_el_ which correspond to those obtained from the absorption measurements, *k*_abs_ (inset of Figure 1b). The apparent values of *k* exceed those of oxide glasses by several orders of magnitude. Also this is a result of the presence of metallic iron inclusions which intrinsically have higher *k* values in accordance with the above observations.

## 3 Conclusion

In summary, we demonstrated record Faraday rotation efficiency in PLD‐derived vitreous films of FeO‐SiO_2_, beating the performance of conventional MO glasses by up to two orders of magnitude. The origin of this is the incorporation of nano‐ and subnanoscopic ferromagnetic clusters of Fe^0^ *_n_*. The formation of these clusters is facilitated by the rapid deposition kinetics occurring during the PLD process and hindering further segregation and growth of precipitates. Due to only weak intercluster interaction, the assembly of FeO‐SiO_2_:Fe^0^ *_n_* exhibits superparamagnetic behavior which is the reason for the unusually strong Faraday effect at room temperature. Superparamagnetic blocking and superspin glass formation occurs in the temperature range of ≈16–32 K, leading to antiferromagnetic interaction between clusters at lower temperature. The presence of metallic Fe was confirmed by XANES and XPS analyses, and also on the complex refractive index of the layers. Using SAED, and HR‐TEM, for the iron orthosilicate thin film, we found cluster sizes of about 1–3 nm.

Of particular interest is the high rotation efficiency of up to 4110 rad m^−1^ achieved in the visible spectral range, i.e., the operation regime of blue, green, and red lasers, and also of super‐continuum light sources. Besides providing the major advantages of superior performance over conventional glassy Faraday rotators, compatibility with virtually any oxide material, optical fiber splicing and fiber device integration, the present approach also presents a general example for creating superparamagnetic vitreous layers with MO functionality. This opens new routes in optical information and laser processing, switching, coding, filtering, and sensing.

## 4 Experimental Section

Amorphous thin films with nominal compositions of *x*FeO·(100 − *x*)SiO_2_ (*x* = 10, 33, 50, 67 in mol%) were deposited on silica glass substrates by PLD. The employed PLD targets were prepared via conventional solid‐state reactions as follows: Reagent‐grade FeO and SiO_2_ were weighed to obtain the prescribed composition, mixed thoroughly, and pelletized. The pellets were sintered at 1000 °C for 10 h in Ar atmosphere for *x* = 67,^31^ and at 1000 °C for 6 h in N_2_ atmosphere for *x* = 10, 33, and 50, respectively. Noteworthy, the choice of inert atmosphere, i.e., Ar or N_2_, does not affect the crystalline phase of the resultant target material. In order to achieve optimal chemical homogeneity of the targets, the procedure of pulverization and resintering was repeated two times in total. The resultant pellets comprised mixtures of Fe_2_SiO_4_, SiO_2_, Fe_3_O_4_, and Fe_2_O_3_ crystalline phases. For each series of material deposition, a PLD target was placed at a distance of 3 cm from the silica substrate. A KrF excimer laser operating at a wavelength of λ = 248 nm and a pulse energy of 180 mJ (10 Hz) was used for target vaporization. Substrates were kept at room temperature in vacuum with a base pressure of 1 × 10^−6^ Pa. For each run, the deposition time was 60 min, resulting in films with a thickness of 260, 360, 280, and 240 nm for *x* = 10, 33, 50, and 67, respectively, as determined with a scanning surface profiler (KLA Tencor Alpha‐Step IQ). With these data, the complex refractive index was modeled from ellipsometric analyses. The chemical composition of the as‐deposited films was verified by Rutherford backscattering spectroscopy using a 2.0 MeV He^2+^‐beam. These analyses yielded actual film compositions of 14.8FeO·85.2SiO_2_, 38.2FeO·61.8SiO_2_, 54.8FeO·45.2SiO_2_, and 67.5FeO·32.5SiO_2_, for the nominals of 10FeO·90SiO_2_, 33FeO·67SiO_2_, 50FeO·50SiO_2_, and 67FeO·33SiO_2_, respectively. Magnetooptical properties and, in particular, Faraday rotation were analyzed at room temperature on a commercial measurement system for Faraday and Kerr effects (JASCO K‐250). For reference, also magnetic properties and the temperature‐dependence of magnetization in a 100 Oe external magnetic field were examined, using a superconducting quantum interference device magnetometer (Quantum Design MPMS‐XL). In order to evaluate the chemical state of iron, the authors carried‐out XPS (ULVAC‐PHI MT‐5500) at room temperature, simultaneously ion sputtering to obtain information in surface and bulk properties of the films (see text for details). The binding energy of the Fe 2p peak was calibrated with the O 1s peak at 529.9 eV.^26^ In parallel, the chemical state of iron was also examined by using XANES measurements on the Fe K‐edge at the BL01B1 beamline of SPring‐8, Hyogo, Japan. The intensity of the XANES spectra was normalized to the edge‐step height and energy after removing the background. The pre‐edge region for each spectrum was integrated to obtain the sums of integrated area. Direct imaging of superparamagnetic iron clusters and particles was performed by TEM using a FEI Tecnai G^2^ FEG operating at 200 kV. Cross‐sections of the films were cut from as‐deposited samples by focused ion beam etching using a FEI Quanta3D FEG dual beam FIB‐SEM workstation. The FIB preparation involved the deposition of a protective Pt layer on the film surface, the thinning with Ga^+^ ions at a high incident angle for minimizing damage, and the sample transfer with an internal micromanipulator. In the present report, the authors focused on analytical TEM data which were obtained for the sample of 67.5FeO·32.5SiO_2_. Additional TEM data for 54.8FeO·45.2SiO_2_ are provided in Figure S1 (Supporting Information). Transmission electron microscopic imaging was done by conventional BF and DF techniques as well as by high‐resolution imaging. SAED was subsequently employed to detect metallic iron particles in the film. The composite dark‐field image was constructed from the maximum gray value of eight DF images of the same area. The underlying DF images were generated by using different parts of a quarter of the ≈2 Å ring in the SAED pattern with the smallest objective aperture of 10 µm.

## Supporting information

As a service to our authors and readers, this journal provides supporting information supplied by the authors. Such materials are peer reviewed and may be re‐organized for online delivery, but are not copy‐edited or typeset. Technical support issues arising from supporting information (other than missing files) should be addressed to the authors.

Supplementary
