# Supplementary material for: Giant Faraday Rotation through Ultrasmall Fe0 n Clusters in Superparamagnetic FeO‐SiO2 Vitreous Films
Source: Adv Sci (Weinh). 2016 Dec 5;4(4):1600299. doi: 10.1002/advs.201600299 (PMC5396158; doi:10.1002/advs.201600299)
Supplement: Supplementary file 1 — Supplementary [file ADVS-4-na-s001.pdf]

Copyright WILEY-VCH Verlag GmbH & Co. KGaA, 69469 Weinheim, Germany, 2016.

ADVANCED SCIENCE
Open Access

Supporting Information

for *Adv. Sci.*, DOI: 10.1002/advs.201600299

Giant Faraday Rotation through Ultrasmall $Fe^0{}_n$ Clusters in Superparamagnetic FeO-SiO_2 Vitreous Films

Yuko Nakatsuka, Kilian Pollok, Torsten Wieduwilt, Falko Langenhorst, Markus A. Schmidt, Koji Fujita, Shunsuke Murai, Katsuhisa Tanaka, and Lothar Wondraczek**

Supporting Information

Giant Faraday rotation through ultra-small Fe^0_n clusters in superparamagnetic FeO-SiO_2 vitreous films

Yuko Nakatsuka, Kilian Pollok, Torsten Wieduwilt, Falko Langenhorst, Markus A. Schmidt, Koji Fujita, Shunsuke Murai, Katsuhisa Tanaka, Lothar Wondraczek**

The SAED pattern of amorphous iron silicate thin film of x = 54.8 is shown in figure S1. No sharp spots or rings are observed in the SAED pattern, indicating that the x = 54.8 thin film is amorphous.

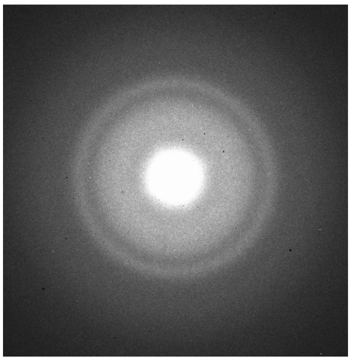

Figure S1. SAED pattern of the x = 54.8 thin film.
